# The Role of Extracellular Vesicles in the Pathogenesis of Metabolic Dysfunction-Associated Steatotic Liver Disease and Other Liver Diseases

**DOI:** 10.3390/ijms26115033

**Published:** 2025-05-23

**Authors:** Elena Grossini, Mohammad Mostafa Ola Pour, Sakthipriyan Venkatesan

**Affiliations:** Laboratory of Physiology, Department of Translational Medicine, Università del Piemonte Orientale, 28100 Novara, Italy; 20046522@studenti.uniupo.it (M.M.O.P.); sakthipriyan.venkatesan@uniupo.it (S.V.)

**Keywords:** alcoholic liver disease, exosomes, microvesicles, mitochondrial dysfunction, pathogenesis, viral hepatitis

## Abstract

The increasing prevalence of liver diseases, such as metabolic dysfunction-associated steatotic liver disease (MASLD), presents considerable medical challenges, particularly given the absence of approved pharmacological treatments, which underscores the necessity to comprehend its underlying mechanisms. Extracellular vesicles (EVs), which are tiny particles released by cells, play a crucial role in facilitating communication and can transport harmful molecules that promote inflammation and tissue damage. These EVs are involved in the progression of various types of liver disorders since they aggravate inflammation and oxidative stress. Because of their critical role, it is believed that EVs are widely involved in the initiation and progression of MASLD, as well as in viral hepatitis, alcoholic liver disease, drug-induced liver injury, and hepatocellular carcinoma. This review emphasizes recent findings regarding the functions of EVs in the above liver pathologies and underscores their potential as new therapeutic targets, paving the way for innovative approaches to address those detrimental liver conditions.

## 1. Introduction

The global rise in obesity and diabetes is closely linked to an increasing prevalence of metabolic dysfunction-associated fatty liver disease (MASLD) and its associated complications. Recent estimates reveal that MASLD affects over one-third of the global population, with a striking prevalence of 50.7% among overweight and obese adults. Notably, the condition is more common in males (59.0%) compared to females (47.5%) [1]. Beyond MASLD, other liver diseases, such as alcoholic liver disease (ALD), viral hepatitis caused by the hepatitis B virus (HBV) or the hepatitis C virus (HCV), and hepatocellular carcinoma (HCC), also represent significant health challenges. ALD affects approximately 20% of heavy drinkers globally, with a higher prevalence in males (30%) than females (10%) [2]. HCC, often arising from chronic liver conditions like cirrhosis, accounts for about 85% of primary liver cancers, with incidence rates varying significantly by regions—higher in East Asia (25 cases per 100,000 individuals) compared to Western countries (5 cases per 100,000 individuals) [3]. Viral hepatitis remains a major public health concern, with HBV affecting approximately 296 million people worldwide and HCV impacting around 58 million individuals, both showing higher prevalence in low- and middle-income countries [4].

MASLD, previously recognized as non-alcoholic fatty liver disease, encompasses a heterogeneous range of liver disorders intricately linked to metabolic dysregulation, characterized by conditions such as obesity, insulin resistance, and dyslipidemia [5]. The pathophysiological framework of MASLD begins with excessive lipid accumulation due to enhanced fatty acid synthesis and impaired fatty acid oxidation, leading to hepatic steatosis—a hallmark of the disease manifested by an excessive deposition of fats within hepatocytes [6,7]. However, a considerable proportion of patients with MASLD progress beyond mere hepatic steatosis to develop non-alcoholic steatohepatitis (NASH), fibrosis, and cirrhosis. Similarly, chronic ethanol exposure in ALD disrupts lipid metabolism, induces oxidative stress, and triggers inflammatory cascades, leading to hepatic steatosis, inflammation, and fibrosis progression [8,9]. In viral hepatitis, viral components may perpetuate chronic inflammation and immune evasion and contribute to fibrogenesis and progression to cirrhosis or HCC [10]. Furthermore, HCC arises predominantly in the context of chronic liver diseases, where persistent oxidative stress, mitochondrial dysfunction, and endoplasmic reticulum (ER) stress would create a favorable microenvironment for tumor growth [11]. As a whole, key factors contributing to MASLD and liver-related diseases include oxidative stress, mitochondrial dysfunction, and ER stress, all of which exacerbate hepatic injury and increase the risk of cardiovascular and metabolic complications [12]. Despite the existing knowledge, a deeper understanding of the above multifactorial mechanisms driving those liver diseases and of the intricate crosstalk between the gut microbiome and liver metabolism is needed to develop effective diagnostic tools and therapeutic strategies to fight the rising burden of liver diseases worldwide [13,14].

Extracellular vesicles (EVs) are gaining attention as key players in liver diseases, facilitating intercellular communication and influencing inflammatory responses and disease progression through the transport of proteins, lipids, and nucleic acids [15]. While other intercellular mediators, such as cytokines, have long been studied for their roles in inflammation and tissue injury, EVs offer unique advantages that make them particularly compelling as biomarkers and therapeutic targets. Unlike soluble cytokines, which often act locally and have short half-lives, EVs are stable, can travel systemically, and carry diverse cargoes—including micro ribonucleic acids (miRNAs), proteins, and lipids—that reflect the molecular signature of their cell of origin [16]. This allows EVs to mediate long-lasting and specific effects on recipient cells, making them critical regulators of both physiological and pathological processes. For instance, emerging evidence suggests that in MASLD, EVs derived from adipose tissue and gut microbiota modulate lipid metabolism, propagate inflammation, and influence fibrosis progression [17]. In ALD, EVs carrying reactive oxygen species (ROS) and pro-inflammatory cytokines amplify liver injury and oxidative stress [18], while in viral hepatitis, they enable immune evasion by transporting viral antigens, sustaining chronic infection [19]. In HCC, EVs drive tumor progression via angiogenesis, immune escape, and metastasis [20,21]. Additionally, the unique protein and ribonucleic acid (RNA) signatures of EVs offer critical insights into disease mechanisms and may serve as prognostic indicators for therapeutic response [22].

The increasing focus on EVs is further driven by recent technological advancements in their isolation and characterization. Techniques such as single-vesicle analysis, nanoparticle tracking analysis (NTA), and multi-omics integration have enabled researchers to gain unprecedented insights into the heterogeneity, cargo composition, and functional properties of EVs [23,24]. These innovations have not only improved the accuracy of EV-based diagnostics but also opened new avenues for engineering EVs as targeted therapeutic agents. For instance, methods like CRISPR/Cas9-based gene editing allow precise manipulation of EVs cargoes, enabling the development of tailored therapies for liver diseases [25]. Such advancements underscore the immense potential of EVs as both diagnostic tools and therapeutic targets, fueling their growing prominence in hepatological research.

This review comprehensively examines the emerging roles of EVs in the pathogenesis of MASLD and broader liver diseases. By elucidating how EVs contribute to lipid metabolism, inflammation, fibrosis, and tumor progression, this review highlights their potential as novel biomarkers and therapeutic targets. Additionally, strategies aimed at modulating EVs secretion or augmenting their therapeutic components could facilitate the development of innovative treatment modalities. The exploration of EVs could not only enhance our understanding of intercellular signaling in the context of liver pathology but could also pave the way for breakthroughs in diagnostics and therapeutics, ultimately improving patient outcomes and advancing our comprehension of liver biology.

## 2. EVs: Biogenesis and Classification

EVs are nano- to microscale membrane-bound structures secreted by virtually all cell types. They serve as critical mediators of intercellular communication by transporting diverse cargoes, including proteins, lipids, nucleic acids, and metabolites. Understanding their biogenesis, classification, and functional roles is paramount for unravelling their contributions to physiological processes and disease pathogenesis.

EVs can be subdivided into exosomes, microvesicles, and apoptotic bodies.

### 2.1. Exosomes: Formation and Functional Diversity

Exosomes are small, membrane-bound vesicles measuring 30–150 nanometers in diameter that originate from the endosomal pathway. Their biogenesis begins with the inward budding of the endosomal membrane, forming intraluminal vesicles (ILVs) within multivesicular bodies (MVBs). These MVBs can either fuse with lysosomes for degradation or merge with the plasma membrane to release ILVs as exosomes into the extracellular space. This process is tightly regulated by the endosomal-sorting complex required for transport (ESCRT), which facilitates the selective packaging of cargo into ILVs [26]. Proteins, such as Alix and Tsg101, also play critical roles in ESCRT-independent pathways, influencing exosome lipid composition and secretion kinetics [27].

Exosomes carry a unique cargo profile, including specific proteins (e.g., cluster of differentiation 63 (CD63), CD81, CD9) and nucleic acids (e.g., miRNAs, messenger RNAs), reflecting the molecular signature of their cells of origin [28]. For instance, hepatocyte-derived exosomes enriched with micro RNA (miR)-122 regulate lipid metabolism and insulin signaling, while those carrying inflammatory cytokines propagate immune responses in MASLD [29]. Their evolutionary conservation underscores their importance in intercellular communication, enabling them to influence target cell behavior through precise cargo delivery.

### 2.2. Microvesicles (MVs): Direct Budding and Pathophysiological Roles

MVs, ranging in size from 100 to 1000 nanometers, bud directly from the plasma membrane through outward blebbing. This process is influenced by cytoskeletal dynamics, calcium levels, and enzymatic activities, such as those of a disintegrin and metalloproteinase domain-containing protein (ADAM) 10 (ADAM10) and ADAM17 [30]. Unlike exosomes, MVs often contain larger fragments of the parent cell’s cytoplasm and organelles, enabling them to deliver more complex signals.

In physiological contexts, MVs contribute to immune regulation, angiogenesis, and coagulation. However, in pathological conditions, such as MASLD, MVs carrying lipid-rich cargoes exacerbate hepatic steatosis and inflammation. For example, adipocyte-derived MVs enriched with ceramides promote insulin resistance and lipotoxicity in hepatocytes [31]. Similarly, Kupffer cell (KC)-derived MVs carrying pro-inflammatory cytokines, such as tumor necrosis factor-alpha (TNF-α) and interleukin 1β (IL-1β), amplify hepatic inflammation and fibrosis progression [32,33]. Emerging evidence suggests that MVs exhibit heterogeneity in size, surface markers, and functional properties, complicating their characterization but also highlighting opportunities for subtype-specific investigations.

### 2.3. Apoptotic Bodies: Markers of Programmed Cell Death

Apoptotic bodies arise during apoptosis, which is a highly regulated form of programmed cell death. As cells undergo apoptosis, nuclear chromatin condenses, the cell membrane blebs outward, and the cellular contents fragment into membrane-enclosed vesicles measuring 500–4000 nanometers in diameter. Unlike exosomes and MVs, apoptotic bodies are uniquely associated with cell death. Actin–myosin interactions drive the blebbing process, leading to the formation of these vesicles, which may vary in size and composition depending on the stage of apoptosis [34].

Efficient clearance of apoptotic bodies by macrophages through phagocytosis is essential for maintaining tissue homeostasis. Key markers of apoptotic bodies include phosphatidylserine, which translocates to the outer leaflet of the cell membrane and serves as an “eat-me” signal recognized by phagocytes via annexin V, thrombospondin, and complement protein C3b. Dysregulated clearance of apoptotic bodies has been implicated in chronic inflammatory conditions, underscoring their relevance in disease pathogenesis [35]. In the context of MASLD, apoptotic bodies derived from injured hepatocytes may propagate inflammation and contribute to fibrogenesis, although their exact role remains under investigation [36].

**Figure 1:**
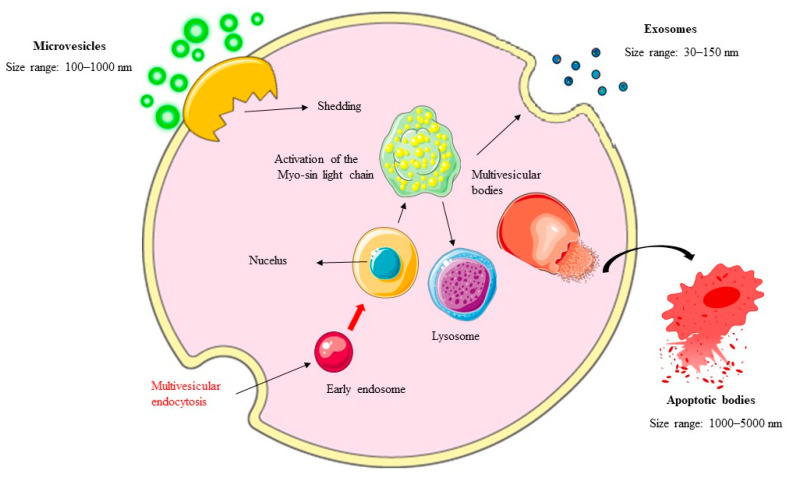
Extracellular vesicles (EVs) biogenesis and secretion. Schematic representation of the origin and release of EVs. Exosomes are formed as intraluminal vesicles by budding into early endosomes. Microvesicles arise as a result of outward budding and fission of the plasma membrane mediated by phospholipid redistribution and cytoskeletal protein contraction. The largest EVs, i.e., apoptotic bodies, are formed during programmed cell death mediated, in part, by actin–myosin mediated membrane blebbing.

In Figure 1, the biogenesis of EVs is depicted.

### 2.4. Other Types of EVs: Ectosomes, Exomeres, and Supermeres

In addition to the well-characterized classes of EVs, such as exosomes, microvesicles, and apoptotic bodies, recent research has identified other types of EVs that further highlight the complexity and heterogeneity in these particles. Among these are ectosomes, exomeres, and supermeres, which add layers of intricacy to our understanding of EVs biogenesis, composition, and function.

Ectosomes, typically ranging in size from 100 to 1000 nanometers, are a distinct class of EVs formed through the outward budding of the plasma membrane, a process that differs significantly from the endosomal trafficking pathways involved in exosome biogenesis. The mechanisms underlying ectosome formation remain poorly understood, but it is clear that the process requires extensive reorganization of both the plasma membrane and cytoskeletal components. This unique biogenesis pathway suggests that ectosomes may carry cargoes and perform functions distinct from those of exosomes or microvesicles, further contributing to the functional diversity of EVs [37]. In addition to vesicular EVs, nonvesicular nanoparticles, such as exomeres and supermeres, have recently been discovered. These particles, typically smaller than 50 nm in diameter, exhibit distinct characteristics compared to traditional small EVs. Exomeres, for instance, display unique proteomic profiles and biodistribution patterns, suggesting specialized roles in intercellular communication and disease progression. Supermeres, on the other hand, are particularly enriched with RNAs and demonstrate enhanced tissue accumulation compared to both exomeres and small EVs [38]. These findings underscore the growing recognition that EVs encompass a broader and more complex spectrum of extracellular particles than previously appreciated.

The identification of these additional EV types highlights the limitations of the current classification framework, which primarily focuses on exosomes, microvesicles, and apoptotic bodies. While this basic framework provides a foundation for understanding EVs biology, it does not fully capture the intricate heterogeneity in and functional diversity of EVs. Further research into the biogenesis, cargo composition, and biological roles of ectosomes, exomeres, and supermeres will be essential to refine our understanding of EVs complexity and its implications in health and disease.

All the information about the heterogeneity in and biogenesis of EVs is mentioned in Table 1.

## 3. MASLD Pathogenesis

**Figure 2:**
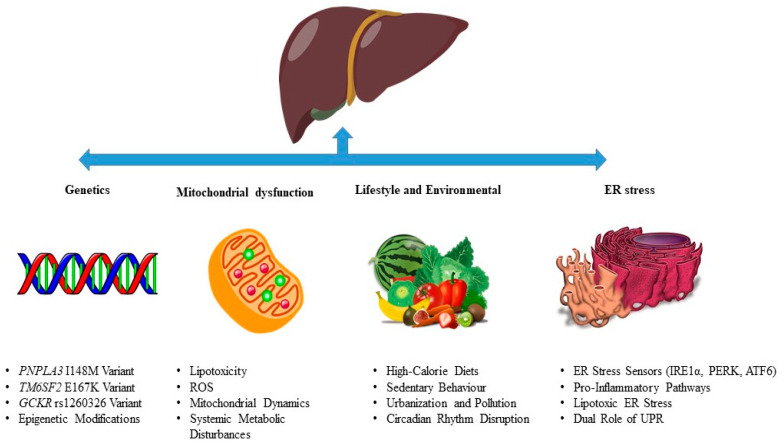
Multifactorial mechanisms underlying metabolic dysfunction-associated fatty liver disease pathogenesis. ATF6: activating transcription factor 6; ER: endoplasmic reticulum; GCKR: glucokinase regulatory protein; IRE1α: inositol-requiring enzyme 1 alpha; PERK: PKR-like endoplasmic reticulum kinase; *PNPLA3*: patatin-like phospholipase domain-containing protein 3; ROS: reactive oxygen species; *TM6SF2*: transmembrane 6 superfamily member 2; UPR: unfolded protein response.

MASLD is a multifactorial disorder driven by genetic predispositions, environmental factors, lifestyle choices, mitochondrial dysfunction, and ER stress (Figure 2). This section delves into the intricate mechanisms underlying MASLD pathogenesis, providing a comprehensive understanding of its progression from simple steatosis to NASH, fibrosis, and cirrhosis.

### 3.1. Genetics in MASLD Pathogenesis

Genetic factors play a pivotal role in determining individual susceptibility to MASLD by modulating lipid metabolism, oxidative stress responses, and hepatic homeostasis. Single nucleotide polymorphisms (SNPs) in genes encoding proteins involved in lipid handling, lipoprotein secretion, and glucose metabolism have been strongly associated with disease risk. These genetic modifiers interact with environmental and lifestyle factors to shape the trajectory of MASLD development, underscoring the importance of understanding their molecular mechanisms for identifying novel therapeutic targets.

One of the most well-characterized genetic determinants of MASLD is the I148M variant (rs738409) in the *patatin-like phospholipase domain-containing protein 3* (*PNPLA3*) gene. *PNPLA3* encodes a protein involved in triacylglycerol hydrolysis, and the I148M substitution reduces its enzymatic activity, impairing lipid breakdown and promoting triglyceride storage in hepatocytes. This variant has been consistently linked to increased hepatic fat accumulation, inflammation, and fibrosis progression in MASLD patients [39]. A meta-analysis by Wang et al. demonstrated that carriers of the I148M variant exhibit a significantly higher risk of severe liver disease, including NASH and cirrhosis, underscoring its clinical relevance [40]. Furthermore, a study by BasuRay et al. revealed that this variant is associated with altered lipid droplet dynamics, exacerbating lipotoxic conditions in genetically predisposed individuals [41].

Another critical genetic determinant is the E167K variant (rs58542926) in the *transmembrane 6 superfamily member 2* (*TM6SF2*) gene. *TM6SF2* encodes a protein involved in lipid droplet trafficking and very-low-density lipoprotein assembly. The E167K substitution impairs very-low-density lipoprotein secretion, leading to reduced lipid export from hepatocytes and increased hepatic fat content. Interestingly, while this variant increases the risk of MASLD, it is associated with lower circulating lipid levels and a reduced risk of cardiovascular disease, as highlighted by O’Hare et al. [42]. This paradox underscores the complexity of *TM6SF2*’s effects on systemic metabolism and highlights the need for personalized interventions targeting this pathway.

Polymorphisms in the *glucokinase regulator* (*GCKR*) gene also contribute to MASLD risk by influencing hepatic glucose metabolism and de novo lipogenesis. The rs1260326 variant, which results in a P446L substitution, alters the activity of glucokinase regulatory protein, leading to increased glucokinase activity and enhanced glucose uptake in hepatocytes [43]. This promotes insulin sensitivity but simultaneously upregulates de novo lipogenesis and may result in excessive lipid synthesis and hepatic fat accumulation. Recent evidence from Wu et al. demonstrated that the rs1260326 variant is associated with altered insulin signaling pathways and elevated triglyceride levels, further linking *GCKR* polymorphisms to MASLD pathogenesis [44].

In addition to these well-established genetic factors, emerging research has identified other SNPs that influence MASLD susceptibility. For instance, variants in the *membrane-bound O-acyltransferase 7 (MBOAT7)* gene, which encodes a lysophosphatidylinositol acyltransferase involved in phospholipid remodeling, have been linked to altered lipid composition in hepatocytes and increased inflammation. Similarly, polymorphisms in *17-beta hydroxysteroid dehydrogenase 13 (HSD17B13)*, which is involved in lipid droplet dynamics, appear to have protective effects against MASLD progression, particularly in reducing inflammation and fibrosis risk. The review by Juanola et al. emphasized the potential of targeting these genetic pathways for therapeutic intervention, as they may offer insights into restoring hepatic lipid homeostasis and mitigating oxidative stress [45].

The interplay between genetic predispositions, environmental factors, and lifestyle choices is crucial in shaping MASLD outcomes. Individuals carrying high-risk variants, such as *PNPLA3 I148M* or *TM6SF2 E167K*, are more susceptible to the adverse effects of high-calorie diets, sedentary behavior, and obesity. Conversely, lifestyle modifications, such as dietary changes and increased physical activity, may mitigate the impact of these genetic risk factors, offering opportunities for personalized prevention strategies. Understanding the precise molecular mechanisms underlying these genetic modifiers remains a priority for future research. For example, delineating how oxidative stress interacts with lipid metabolism disruption in genetically predisposed individuals could reveal novel therapeutic targets [46].

Recent studies have expanded our understanding of the functional consequences of these genetic variants. BasuRay et al. showed that the I148M variant impairs the ability of *PNPLA3* to catalyze triacylglycerol hydrolysis, leading to intracellular lipid accumulation and impaired autophagy [41]. Similarly, investigations by Eslam et al. revealed that the E167K variant disrupts the interaction between *TM6SF2* and microsomal triglyceride transfer protein, altering lipid export pathways and contributing to hepatic steatosis [47]. These findings highlight the importance of integrating genetic data with functional studies to develop genotype-guided interventions for MASLD management.

Moreover, epigenetic modifications influenced by genetic variants may also contribute significantly to MASLD pathogenesis. For instance, deoxyribonucleic acid (DNA) methylation patterns near the *PNPLA3* locus have been shown to regulate its expression levels, potentially modifying the penetrance of the I148M variant, which is strongly associated with hepatic fat accumulation and inflammation [48]. Similarly, histone acetylation at the promoter region of *GCKR* enhances its transcriptional activity, exacerbating de novo lipogenesis in response to high-carbohydrate diets, thereby promoting excessive lipid synthesis and hepatic steatosis [49]. Beyond these examples, recent studies have highlighted the role of global DNA hypomethylation in altering the expression of genes involved in lipid metabolism and inflammation, further amplifying metabolic dysfunction in MASLD [50]. Additionally, histone modifications, such as H3K27 acetylation, have been linked to the upregulation of pro-inflammatory cytokines like TNF-α and interleukin 6 (IL-6), contributing to chronic liver inflammation and fibrosis progression [51]. These findings underscore the intricate interplay between genetic predispositions, epigenetic regulation, and metabolic pathways in MASLD, emphasizing the need for further research to unravel these complex interactions and identify novel therapeutic targets.

The summary of various EVs genetic cargoes involved in MASLD is depicted in Table 2. 

### 3.2. Lifestyle and Environmental Influences in MASLD Pathogenesis

Lifestyle and environmental factors are pivotal contributors to the development and progression of MASLD. The global rise in MASLD prevalence is closely tied to modern lifestyle changes, including high-calorie diets, sedentary behavior, urbanization, and exposure to environmental toxins. These factors collectively disrupt metabolic homeostasis, leading to hepatic fat accumulation, insulin resistance, systemic inflammation, and, ultimately, liver injury.

High-calorie diets, particularly those rich in saturated fats, refined carbohydrates, and fructose, are strongly associated with the onset of MASLD. Such dietary patterns, which are prevalent in Western countries, promote excessive caloric intake and disrupt lipid metabolism. Excessive fructose consumption, for instance, has been shown to upregulate de novo lipogenesis in hepatocytes, leading to increased triglyceride synthesis and hepatic steatosis [52]. Regarding this issue, Muriel et al. demonstrated that high-fructose diets can exacerbate mitochondrial dysfunction and promote oxidative stress in the liver, further contributing to disease progression [53]. As reported above, it should be, however, highlighted that genetic predispositions could increase the predisposition to develop MASLD. In particular, subjects carrying variants in the *PNPLA3* or *TM6SF2* genes are more vulnerable to the adverse effects of high-fat diets, as these genetic factors impair lipid export and increase susceptibility to hepatic fat accumulation [47].

Physical inactivity is another critical environmental factor driving MASLD pathogenesis. Sedentary behavior reduces energy expenditure and promotes adipose tissue expansion, which, in turn, increases the release of pro-inflammatory cytokines and free fatty acids (FFAs) into the circulation. FFAs are taken up by hepatocytes, where they undergo esterification to form triglycerides, which are hallmarks of hepatic steatosis. Furthermore, physical inactivity exacerbates insulin resistance, creating a feedback loop that perpetuates metabolic dysfunction [54]. A study by Sargeant et al. highlighted that regular aerobic exercise significantly reduces hepatic fat content and improves insulin sensitivity, underscoring the importance of physical activity in MASLD prevention and management [55]. The “double-hit hypothesis” provides a foundational framework for understanding the progression of MASLD. According to this model, the first “hit” involves insulin resistance, which initiates hepatic steatosis through mechanisms such as increased lipolysis in adipose tissue and enhanced de novo lipogenesis in the liver. Insulin resistance leads to activated lipolysis in adipose tissue, resulting in the release of FFAs into the bloodstream. These FFAs are absorbed in the liver and undergo de novo lipogenesis, resulting in the accumulation of triglycerides in hepatocytes. The second “hit” encompasses additional stressors, including mitochondrial dysfunction, ER stress, oxidative stress, and chronic inflammation, which collectively drive the transition from simple steatosis to NASH. Recent evidence suggests that this model may be oversimplified, as multiple parallel hits—such as gut dysbiosis, circadian rhythm disruption, and epigenetic modifications—contribute to disease progression, as well [56].

Urbanization has introduced additional environmental stressors, such as exposure to endocrine-disrupting chemicals and air pollution, which may contribute to MASLD pathogenesis. Endocrine-disrupting chemicals, including bisphenol A and phthalates, have been shown to disrupt lipid metabolism and promote insulin resistance. Similarly, chronic exposure to fine particulate matter has been associated with increased systemic inflammation and oxidative stress, both of which exacerbate liver injury. Cheng et al. revealed that individuals living in highly polluted urban areas had a higher prevalence of MASLD compared to those in rural regions, highlighting the impact of environmental factors on disease risk [57].

Circadian rhythm disruption, often caused by irregular sleep patterns, shift work, or jet lag, has emerged as a novel contributor to MASLD pathogenesis. The liver exhibits circadian regulation of metabolic processes, and disruption of these rhythms may lead to impaired glucose and lipid metabolism. For instance, clock genes, such as *brain and muscle Arnt-like protein-1* and *circadian locomotor output cycles kaput,* regulate hepatic lipid synthesis and oxidation, and their dysregulation contributes to hepatic steatosis and inflammation [58]. Recent studies have shown that time-restricted feeding—a strategy aimed at restoring circadian alignment—can improve insulin sensitivity and reduce hepatic fat accumulation in preclinical models of MASLD [59].

Sex hormones also play a significant role in MASLD pathogenesis, with estrogen offering protection against hepatic fat accumulation and inflammation in women, particularly during reproductive years. Postmenopausal women, instead, lose this protective effect because of reduced estrogen levels, making them more susceptible to MASLD. Testosterone, on the other hand, promotes visceral adiposity and insulin resistance in men, contributing to a higher prevalence of MASLD in males compared to females [60]. Emerging research by Kim et al. suggests that hormonal therapies, such as estrogen replacement, may alleviate MASLD in postmenopausal women, although long-term safety concerns remain [61].

### 3.3. Mitochondrial Dysfunction in MASLD Pathogenesis

Mitochondrial dysfunction is a central driver of MASLD, contributing to impaired energy metabolism, oxidative stress, and inflammation. As the primary site of fatty acid oxidation and adenosine triphosphate (ATP) production, mitochondria are essential for maintaining hepatic metabolic homeostasis. However, excessive lipid influx in MASLD overwhelms mitochondrial capacity, leading to reduced fatty acid oxidation and the accumulation of toxic lipid intermediates, such as diacylglycerols and ceramides. This lipotoxicity, in turn, impairs insulin signaling and promotes hepatocyte injury.

Excessive lipid accumulation in hepatocytes disrupts mitochondrial function by overwhelming β-oxidation pathways and increasing ROS production. ROS damage cellular components, including mitochondrial DNA, proteins, and lipids, creating a vicious cycle of oxidative stress and mitochondrial dysfunction. Dysregulation of mitochondrial dynamics—such as excessive fission mediated by dynamin-related protein 1 (Drp1) or impaired mitophagy—further compromises mitochondrial quality and function, amplifying liver injury. For example, Zhang et al. demonstrated that Drp1-mediated mitochondrial fission exacerbates ROS production and promotes apoptosis in hepatocytes, highlighting its role in MASLD progression [62].

Mitochondrial dysfunction also contributes to systemic metabolic disturbances by impairing gluconeogenesis and urea cycle activity. Reduced ATP production due to mitochondrial damage limits the liver’s ability to maintain energy homeostasis, exacerbating insulin resistance and dyslipidemia [63]. A review by Zhao et al. emphasized the importance of preserving mitochondrial function to restore hepatic metabolic balance in MASLD patients [64]. Strategies aimed at enhancing mitochondrial biogenesis, such as pharmacological activation of peroxisome proliferator-activated receptor gamma coactivator 1-alpha, have shown promise in preclinical models of MASLD [65].

Furthermore, mitochondrial dysfunction interacts with other cellular stressors, such as ER stress and oxidative stress, creating a synergistic effect that drives disease progression. Crosstalk between mitochondria and the ER has been implicated in lipotoxicity-induced apoptosis, as damaged mitochondria would release cytochrome c, triggering caspase activation and cell death. Recent evidence suggests that targeting mitochondrial quality control pathways, such as mitophagy or fusion–fission balance, offers promising therapeutic potential for MASLD [66]. Jiang et al. demonstrated that elamipretide, which is a mitochondrial-targeted peptide, improves hepatic mitochondrial function and reduces markers of inflammation in MASLD patients, underscoring the translational relevance of this approach [67].

### 3.4. ER Stress and the Unfolded Protein Response (UPR) in MASLD Pathogenesis

ER stress and UPR are critical mechanisms underlying the progression of MASLD, particularly in the transition from simple steatosis to NASH. Excessive lipid accumulation in hepatocytes disrupts ER homeostasis, overwhelming its capacity to properly fold proteins and triggering the activation of the UPR. This adaptive response involves three key ER stress sensors—inositol-requiring enzyme 1 alpha (IRE1α), protein kinase RNA- like endoplasmic reticulum kinase (PERK), and activating transcription factor 6—which initially aim to restore proteostasis but, when chronically activated, promote inflammation, apoptosis, and oxidative stress.

**Figure 3:**
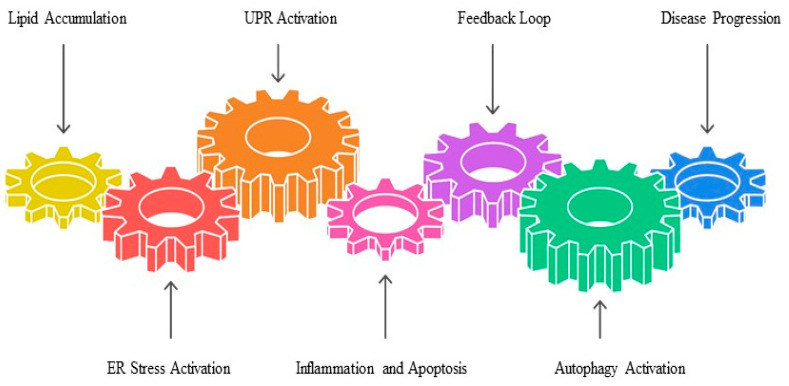
Endoplasmic reticulum stress and unfolded protein response in metabolic dysfunction-associated fatty liver disease progression. ER: endoplasmic reticulum; UPR: unfolded protein response.

Chronic ER stress exacerbates hepatic injury by activating pro-inflammatory pathways. For instance, IRE1α signaling activates c-Jun N-terminal kinase (JNK) and nuclear factor-kappa B (NF-κB), propagating inflammatory cascades that amplify liver damage. Similarly, PERK activation induces oxidative stress through increased ROS production and impairs insulin signaling, creating a feedback loop of metabolic dysfunction and cellular injury. Cao et al. demonstrated that IRE1α-mediated JNK activation significantly enhances TNF-α production in KCs, exacerbating hepatic inflammation in MASLD patients [68]. ER stress also contributes to lipotoxicity by altering the composition of the lipid bilayer, leading to membrane instability and dysfunction. Lipotoxic ER stress arises when excess FFAs enter the ER lumen, disrupting its structural integrity and impairing protein folding capacity. Recent evidence suggests that specific FFAs, such as palmitate, are particularly potent inducers of ER stress in hepatocytes [69]. This process is closely linked to mitochondrial dysfunction, as damaged mitochondria generate ROS that further exacerbate ER stress, creating a vicious cycle of cellular damage (Figure 3).

Emerging research highlights the dual role of ER stress in either promoting or mitigating disease progression. For example, transient activation of the UPR can induce autophagy, which is a protective mechanism that clears misfolded proteins and restores ER function. However, prolonged ER stress leads to sustained inflammation and apoptosis, driving fibrogenesis and cirrhosis [70].

## 4. The Role of EVs in MASLD Pathogenesis

EVs have emerged as critical mediators in the pathogenesis of MASLD. By facilitating intercellular communication, EVs transport bioactive molecules, such as lipids, proteins, miRNAs, and metabolites that influence lipid metabolism, inflammation, fibrosis, and gut–liver crosstalk. Understanding the specific roles of EVs in these processes provides valuable insights into novel diagnostic and therapeutic strategies for managing MASLD.

### 4.1. Lipid Metabolism

One of the primary mechanisms through which EVs influence lipid metabolism is by modulating de novo lipogenesis in hepatocytes. For instance, EVs derived from adipose tissue carry miRNAs, such as miR-122 and miR-34a, which upregulate enzymes involved in fatty acid synthesis, including fatty acid synthase and acetyl-CoA carboxylase. This promotes excessive triglyceride accumulation in hepatocytes. In the study by Zhou et al., it was demonstrated that adipocyte-derived EVs enriched with specific lipid cargoes directly enhance de novo lipogenesis in recipient hepatocytes, highlighting the role of EVs in lipid dysregulation during MASLD progression [71,72].

In addition to promoting lipogenesis, EVs also impair fatty acid oxidation, further contributing to lipid accumulation. Mitochondrial dysfunction, a well-established feature of MASLD, is exacerbated by EVs carrying pro-inflammatory cytokines or miRNAs that downregulate genes involved in β-oxidation. For example, EVs containing miR-192 have been linked to the reduced expression of carnitine palmitoyltransferase 1, a critical enzyme in mitochondrial fatty acid oxidation [73,74]. This disruption in lipid metabolism creates a feedback loop of lipotoxicity, oxidative stress, and inflammation, driving the transition from simple steatosis to more severe forms of liver injury. Recent evidence by Heida et al. suggests that this process is closely linked to the activation of NF-κB-related signaling in hepatocytes [75]. Adipose tissue-derived EVs not only transport lipids but also signal to the liver to modulate insulin sensitivity and lipid handling. For example, EVs enriched with ceramides and diacylglycerols impair insulin signaling in hepatocytes, exacerbating hepatic steatosis and metabolic dysfunction [76]. Furthermore, the interaction between EVs and hepatic lipid droplets has been implicated in shaping the intracellular environment conducive to fat accumulation. Cho et al. highlighted that adipose tissue-derived EVs carrying specific lipids alter lipid droplet dynamics, contributing to lipotoxic conditions in genetically predisposed individuals [77].

Recent studies have expanded our understanding of the functional diversity of EVs in lipid metabolism. For instance, Li et al. demonstrated that EVs derived from injured hepatocytes carry lipid-rich cargoes that disrupt phospholipid bilayer integrity, leading to membrane instability and impaired protein trafficking. Additionally, the heterogeneity in EV populations derived from different cell types underscores their specialized roles in lipid metabolism. For example, EVs from KCs carry distinct lipid profiles compared to those from hepatocytes, influencing hepatic lipid uptake and export pathways differently, as well [78]. Emerging research also emphasizes the importance of EV cargo sorting mechanisms in determining their effects on lipid metabolism. The ESCRT machinery and Ras-associated binding guanine triphosphatases regulate the incorporation of specific lipids and miRNAs into EVs, ensuring precise delivery to target cells. Dysregulation of these pathways in MASLD leads to altered EVs composition, promoting hepatic steatosis and metabolic imbalance. Chen et al. demonstrated that inhibiting ESCRT-dependent pathways reduced the transfer of pro-lipogenic cargoes via EVs, underscoring its potential as a preventive strategy [79].

The dynamic interplay between EVs and systemic lipid metabolism highlights their relevance in disease progression. For example, EVs derived from adipocytes under conditions of insulin resistance carry elevated levels of FFAs and inflammatory mediators, creating a feedback loop of metabolic dysfunction and cellular injury [80]. Moreover, the interaction between EVs and hepatic lipid sensors, such as sterol regulatory element-binding proteins-1c and peroxisome proliferator-activated receptor α (PPARα), modulates the transcriptional activity of genes involved in lipid synthesis and oxidation [81].

### 4.2. Inflammation

Inflammation is a central driver of MASLD progression, and EVs have emerged as key mediators of inflammatory signaling in the liver. These vesicles transport pro-inflammatory cargoes, including cytokines, miRNAs, and lipids, amplifying immune responses and promoting hepatocyte injury. Understanding the role of EVs in inflammation provides critical insights into the mechanisms underlying MASLD pathogenesis.

KCs, the resident macrophages of the liver, are a primary source of pro-inflammatory EVs. KCs release EVs enriched with pro-inflammatory cytokines, like TNF-α, IL-6 and IL-1β. In this way, EVs propagate inflammatory signals to neighboring hepatocytes, hepatic stellate cells (HSCs), and other immune cells, creating a cascade of liver inflammation [82]. For instance, Babuta et al. demonstrated that KC-derived EVs carrying miR-155 may activate the NF-κB pathway in hepatocytes, exacerbating inflammation and contributing to the transition from simple steatosis to NASH [83].

A key mechanism related to EVs involves the activation of inflammasomes, which are multiprotein complexes responsible for producing pro-inflammatory cytokines, such as IL-1β. EVs containing RING finger protein 213 have been found to intensify nucleotide-binding domain, leucine-rich-containing family, and pyrin domain-containing-3-inflammasome activation. This leads to hepatocyte pyroptosis—a type of programmed cell death associated with severe liver inflammation [84]. According to Wang et al., this process is intricately connected to oxidative stress and mitochondrial dysfunction, further exacerbating inflammatory signaling pathways [40].

The recruitment and polarization of immune cells also play a significant role in EV-mediated inflammation. For example, EVs derived from adipose tissue carry chemokines, like monocyte chemoattractant protein-1, which recruit circulating monocytes to the liver [85]. These monocytes differentiate into pro-inflammatory macrophages, perpetuating the cycle of inflammation and fibrosis. Recent studies have shown that the composition of EV cargoes varies depending on the stage of MASLD, with advanced stages characterized by increased levels of pro-inflammatory cytokines and ROS-inducing molecules [86].

Emerging research highlights the dual role of EVs in either promoting or mitigating inflammation. For instance, EVs derived from regulatory T cells carry anti-inflammatory factors, such as interleukin-10 and transforming growth factor-beta (TGF-β), which suppress excessive immune activation [87]. However, under pathological conditions, the balance shifts toward pro-inflammatory EVs, driven by factors such as oxidative stress and genetic predispositions. Restoring this balance through pharmacological or genetic interventions may represent a promising avenue for managing MASLD-related inflammation.

**Figure 4:**
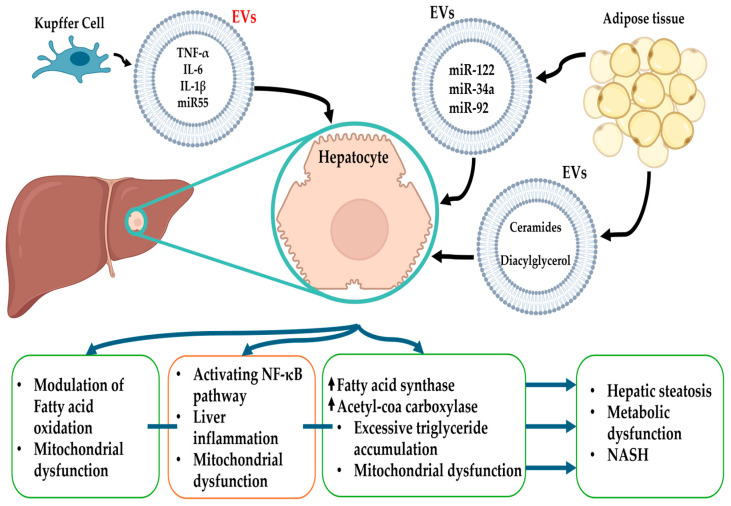
Role of extracellular vesicles in metabolic dysfunction-associated fatty liver disease pathogenesis and important cargoes. EVs: extracellular vesicles; IL: interleukin; NASH: non-alcoholic fatty liver disease; NF-κB: nuclear factor kappa-light-chain-enhancer of activated B cells; miR: microRNA; TNF-α: tumor necrosis factor-alpha; ↑: Increase.

The interaction between EVs and systemic inflammatory networks underscores their significance in disease progression. For example, EVs carrying damage-associated molecular patterns (DAMPs), such as high mobility group box 1 and heat shock proteins, amplify innate immune responses in the liver microenvironment [88]. Additionally, the heterogeneity in EVs populations derived from different immune cells (e.g., neutrophils, dendritic cells) influences the magnitude and duration of inflammatory cascades [89]. Recent evidence suggests that targeting specific EV cargoes, such as DAMPs or miR-155, could mitigate chronic inflammation in MASLD patients. The role of EV cargoes in the pathophysiology of MASLD is summarized in Figure 4.

### 4.3. Fibrosis and Remodeling

Fibrosis represents a critical turning point in the progression of MASLD, marking the transition from reversible hepatic steatosis to potentially irreversible liver damage. EVs have emerged as key players in mediating fibrogenesis, facilitating crosstalk between hepatocytes, immune cells, and HSCs that perpetuates extracellular matrix (ECM) remodeling and tissue scarring.

A central mechanism through which EVs could contribute to fibrosis is the activation of HSCs, the primary effector cells responsible for ECM production. EVs released by injured hepatocytes, KCs, and other liver-resident cells carry profibrotic cargoes such as TGF-β, connective tissue growth factor, and miRNAs (e.g., miR-21, miR-199a). These molecules induce HSC trans-differentiation into myofibroblast-like cells, which secrete excessive amounts of collagen and other ECM proteins, disrupting liver architecture [90]. Povero et al. demonstrated that hepatocyte-derived EVs enriched with TGF-β are able to significantly enhance HSCs activation and collagen deposition in rodent models of MASLD, underscoring their pivotal role in fibrosis progression [91].

In addition to activating HSCs, EVs also influence ECM turnover by modulating the balance between matrix-degrading enzymes and their inhibitors. For instance, EVs carrying matrix metalloproteinases (MMPs) and tissue inhibitors of metalloproteinases (TIMPs) regulate ECM degradation and synthesis. Dysregulation of this balance, often mediated by profibrotic EVs, leads to excessive ECM accumulation and impaired tissue repair [92]. Recent evidence suggests that targeting EV-mediated MMP/TIMP signaling could restore ECM homeostasis and mitigate fibrosis severity, as highlighted by Zhang et al. [93]. Chronic oxidative stress and mitochondrial dysfunction further exacerbate fibrosis by amplifying the release of profibrotic EVs. For example, EVs derived from hepatocytes under oxidative stress transport ROS-inducing molecules that sustain HSCs activation and ECM remodeling [78]. Furthermore, crosstalk between EVs and mitochondrial dysfunction can create a feedback loop that perpetuates ROS production, lipotoxicity, and ECM deposition. This interaction is particularly relevant in advanced stages of MASLD, where persistent liver injury drives aberrant wound-healing responses [94].

The functional diversity of EVs in fibrosis progression is also influenced by their origin and cargo composition. For instance, EVs derived from activated KCs carry distinct profibrotic signatures compared to those from quiescent hepatocytes, reflecting the dynamic nature of EV-mediated signaling. Recent advances in single-vesicle analysis allow for detailed characterization of these subtypes, enabling subtype-specific interventions tailored to individual disease stages [83]. Additionally, the interaction between EVs and ECM components, such as collagens and fibronectins, shapes the mechanical properties of the liver microenvironment, influencing cell behavior and disease progression [95].

Emerging research has identified novel pathways through which EVs could contribute to fibrogenesis. For example, EVs carrying integrins and adhesion molecules may facilitate the attachment of HSCs to ECM scaffolds, enhancing their activation and proliferation. Similarly, the transfer of specific lipids via EVs could alter membrane fluidity in recipient cells, promoting fibrotic signaling pathways [92]. These findings highlight the complexity of EV-mediated mechanisms in MASLD fibrosis and emphasize the need for comprehensive investigations into their functional consequences.

### 4.4. Gut Microbiota and EVs

The gut microbiota plays a critical role in MASLD pathogenesis, and EVs derived from gut microbiota have emerged as key mediators of gut–liver crosstalk. These microbiota-derived EVs transport bacterial components, metabolites, and signaling molecules to the liver via the portal circulation, influencing hepatic lipid metabolism, inflammation, and fibrosis.

**Figure 5:**
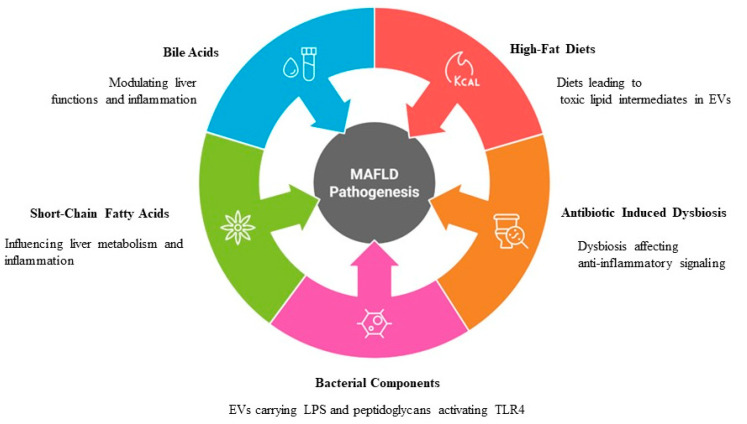
Role of gut microbiota-derived extracellular vesicles in metabolic dysfunction-associated fatty liver disease. EVs: extracellular vesicles; MASLD: metabolic associated steatotic liver disease; LPS: lipopolysaccharide; TLR4: toll-like receptor 4.

Gut microbiota-derived EVs transport lipopolysaccharides (LPSs), peptidoglycans, and microbial metabolites to the liver, where they modulate immune responses and metabolic pathways. For instance, EVs carrying LPSs activate Toll-like receptor 4 (TLR4) signaling in KCs and hepatocytes, triggering pro-inflammatory cascades and exacerbating liver injury [96]. This process is closely linked to increased intestinal permeability (“leaky gut”), which allows bacterial products to enter systemic circulation and amplify hepatic inflammation [97]. Papadakos et al. demonstrated that gut microbiota-derived EVs enriched with LPSs significantly upregulate TLR4-mediated NF-κB signaling in the liver, highlighting their role in perpetuating chronic inflammation during MASLD progression (Figure 5) [98].

In addition to LPSs, microbiota-derived EVs also carry short-chain fatty acids and bile acids, which influence hepatic lipid metabolism and insulin sensitivity. Short-chain fatty acids, such as butyrate and propionate, are known for their anti-inflammatory properties; however, dysbiosis in MASLD alters the composition of these EV cargoes, reducing their protective effects [99]. Similarly, altered bile acid metabolism mediated by gut microbiota-derived EVs disrupts farnesoid X receptor (FXR) and Takeda G protein-coupled receptor 5 signaling (TGR5), contributing to hepatic steatosis and fibrosis [100]. Díaz-Garrido et al. emphasized the dual role of microbiota-derived EVs in either promoting or mitigating liver disease, depending on the specific cargo and microbial community composition [101]. Furthermore, gut microbiota-derived EVs could contribute to systemic oxidative stress and mitochondrial dysfunction. For example, EVs carrying pro-inflammatory cytokines or ROS-inducing molecules would exacerbate mitochondrial damage in hepatocytes, creating a vicious cycle of oxidative stress and lipotoxicity [102]. Recent studies have shown that targeting these EV-mediated pathways offers promising potential for MASLD management. Pharmacological interventions aimed at modulating gut microbiota composition—such as prebiotics, probiotics, or fecal microbiota transplantation—can alter the production and cargo composition of microbiota-derived EVs, thereby restoring metabolic homeostasis in the liver [103].

The functional diversity of gut microbiota-derived EVs is influenced by dietary patterns, antibiotic use, and host–microbiota interactions. For instance, high-fat diets alter the composition of gut bacteria, leading to the release of EVs enriched with toxic lipid intermediates, such as ceramides and diacylglycerols [104]. Similarly, antibiotic-induced dysbiosis reduces the secretion of short-chain fatty acid-carrying EVs, impairing anti-inflammatory signaling in the liver [105]. These findings underscore the importance of maintaining a healthy gut microbiota to prevent MASLD progression.

Probiotics, another class of dietary intervention, also influence microbiota-derived EV profiles by introducing beneficial bacterial strains that secrete anti-inflammatory EVs. For instance, probiotic strains like Lactobacillus rhamnosus and Bifidobacterium longum release EVs carrying anti-inflammatory miRNAs, such as miR-124 and miR-223, which suppress NF-κB signaling and reduce cytokine production in the liver microenvironment [106]. Additionally, probiotic-derived EVs enhance intestinal barrier integrity by upregulating tight junction proteins like occludin and zonula occludens-1, thereby reducing intestinal permeability and limiting the translocation of pro-inflammatory EVs to the liver [107]. Recent studies have highlighted the potential of combining dietary fiber with pharmacological agents to optimize microbiota-derived EV profiles. Soluble fibers, such as pectin and beta-glucan, are fermented by gut bacteria to produce short chain fatty acids, which are subsequently packaged into EVs. These short chain fatty acids-enriched EVs regulate hepatic lipid metabolism by activating FXR and TGR5 signaling pathways, both of which are critical for maintaining bile acid homeostasis and preventing hepatic steatosis [108]. Conversely, low-fiber diets lead to dysbiosis and the release of EVs enriched with toxic lipid intermediates, such as ceramides and diacylglycerols, which exacerbate lipotoxicity and oxidative stress in the liver [109].

Emerging research highlights the potential of engineering microbiota-derived EVs for targeted interventions in MASLD. For example, EVs engineered to deliver anti-inflammatory miRNAs, such as miR-124 or miR-223, can suppress NF-κB signaling and reduce cytokine production in the liver microenvironment [110]. Additionally, blocking the secretion of pro-inflammatory EVs by using pharmacological inhibitors of biogenesis pathways has been shown to alleviate liver injury in rodent models of NASH [86]. These strategies offer innovative approaches for managing gut–liver axis dysfunction in MASLD.

## 5. Extracellular Vesicles in Other Liver Diseases

EVs play a critical role in the pathogenesis of various liver diseases beyond MASLD. This section explores the specific contributions of EVs to viral hepatitis, ALD, drug-induced liver injury (DILI), and HCC. Understanding these mechanisms provides valuable insights into the broader role of EVs in liver biology and disease progression.

### 5.1. EVs in Viral Hepatitis

Viral hepatitis, including infections caused by HBV and HCV, represents a significant global health burden. EVs have emerged as key mediators in the pathogenesis of these conditions, facilitating viral replication, immune evasion, and liver injury. By transporting viral components, proteins, miRNAs, and metabolites, EVs influence both local and systemic responses during viral hepatitis.

One of the primary mechanisms through which EVs can contribute to viral hepatitis is by promoting viral replication and dissemination. Both HBV and HCV hijack the host cell’s EVs biogenesis machinery to package viral components, such as viral RNA and proteins, into EVs. These EVs then serve as vehicles for transferring viral material to neighboring hepatocytes, amplifying infection within the liver microenvironment.

Notably, recent studies have highlighted the dual roles of EVs in liver disease progression. For instance, Jiang et al. demonstrated that enhancing hepatic EVs secretion from steatotic hepatocytes exacerbates inflammation and fibrosis, worsening disease outcomes [111]. Furthermore, Kouroumalis et al. demonstrated that HCV-infected hepatocytes release EVs enriched with viral RNA, which are internalized by uninfected cells and promote de novo viral replication [112]. Similarly, HBV exploits EV-mediated pathways to shield viral antigens from immune surveillance, sustaining chronic infection even in the presence of antiviral therapies [113].

In addition to facilitating viral replication, EVs modulate the host immune response during viral hepatitis. Infected hepatocytes release EVs carrying immunomodulatory molecules, such as programmed death-ligand 1 (PD-L1) and suppressor of cytokine signaling proteins, which dampen antiviral immunity. Zhang et al. highlighted how HCV-derived EVs suppress interferon signaling in immune cells, impairing their ability to mount an effective antiviral response [114]. Furthermore, EVs carrying pro-inflammatory cytokines exacerbate liver injury by recruiting immune cells to the liver and perpetuating inflammatory cascades. Recent evidence suggests that neutralizing DAMPs-enriched EVs could mitigate excessive inflammation and improve outcomes in affected patients [115].

The functional diversity of EVs in viral hepatitis underscores their importance in disease progression. For example, EVs derived from KCs carry distinct cargoes compared to those from infected hepatocytes, influencing immune activation and fibrogenesis differently [116]. Additionally, chronic inflammation driven by EV-mediated immune dysregulation contributes to fibrosis development, where TGF-β-enriched EVs activate HSCs and promote ECM deposition [117]. Future studies should focus on unraveling the precise mechanisms governing EV-mediated processes in viral hepatitis and evaluating their translational potential in clinical settings.

### 5.2. EVs in ALD

ALD encompasses a spectrum of conditions ranging from simple steatosis to alcoholic hepatitis, fibrosis, and cirrhosis, all driven by chronic ethanol consumption. EVs significantly contribute to ALD pathogenesis by mediating oxidative stress, inflammation, gut–liver crosstalk, and hepatocyte apoptosis. Their ability to transport bioactive molecules reflects the dynamic interplay between environmental factors and cellular responses during ALD progression.

A primary mechanism by which EVs exacerbate ALD is through the propagation of oxidative stress and mitochondrial dysfunction. Chronic ethanol exposure induces the production of ROS in hepatocytes, leading to cellular damage and impaired mitochondrial function. EVs derived from ethanol-stressed hepatocytes carry ROS-inducing molecules that further amplify oxidative stress in recipient cells, creating a feedback loop of hepatocyte injury [118]. Eguchi et al. demonstrated that hepatocyte-derived EVs enriched with ROS significantly enhanced mitochondrial dysfunction and apoptosis in rodent models of ALD [119].

Inflammation is another hallmark feature of ALD, and EVs play a pivotal role in amplifying this process. Ethanol metabolism generates toxic intermediates, such as acetaldehyde, which activate KCs and other immune cells, triggering the release of pro-inflammatory cytokines, such as TNF-α, IL-6, and IL-1β. These cytokines are packaged into EVs, which propagate inflammatory signals throughout the liver microenvironment [120]. Recent evidence suggests that EVs carrying TNF-α and IL-6 enhance the recruitment of neutrophils and macrophages to the liver, exacerbating tissue injury [121].

The gut–liver axis plays a crucial role in ALD pathogenesis, and gut microbiota-derived EVs significantly contribute to this interaction. Ethanol-induced disruption of the intestinal barrier increases intestinal permeability (“leaky gut”), allowing bacterial products such as LPSs to enter systemic circulation via EV-mediated transport [122]. Once in the liver, LPS-enriched EVs activate TLR4 signaling in KCs and hepatocytes, triggering pro-inflammatory cascades and perpetuating liver injury [123]. Dysbiosis in ALD alters the composition of microbiota-derived EV cargoes, reducing their protective effects and promoting pathogenic interactions [124].

Hepatocyte apoptosis represents another key feature of ALD progression, facilitated by EVs carrying death ligands, such as Fas ligand and tumor necrosis factor-related apoptosis-inducing ligand. These EVs induce apoptosis in recipient hepatocytes, contributing to progressive liver dysfunction and fibrosis. Crosstalk between EVs and HSCs amplifies fibrogenesis by activating ECM remodeling pathways, underscoring the multifaceted role of EVs in ALD pathophysiology [125].

Emerging research highlights the heterogeneity in EVs populations in ALD, suggesting specialized functions depending on their origin and cargo composition. For instance, EVs derived from activated KCs carry distinct pro-inflammatory signatures compared to those from injured hepatocytes, reflecting the complex interplay between innate immunity and hepatocyte injury. Advances in single-vesicle analysis allow for detailed characterization of these subtypes, paving the way for subtype-specific interventions tailored to individual disease stages [126].

### 5.3. EVs in DILI

DILI poses a significant challenge in clinical practice due to its unpredictable nature and association with severe complications. EVs contribute to DILI pathogenesis by mediating drug metabolism, propagating hepatotoxicity, and modulating immune responses. Their ability to transport specific cargoes enables them to influence both local and systemic effects during DILI. Acetaminophen overdose, a common cause of acute liver failure, induces the release of EVs carrying N-acetyl-p-benzoquinone imine, a highly reactive metabolite responsible for hepatotoxicity. These EVs transfer N-acetyl-p-benzoquinone imine to unexposed hepatocytes, propagating oxidative stress and mitochondrial dysfunction. Umbaugh et al. demonstrated that inhibiting EVs secretion using pharmacological agents reduced the spread of acetaminophen-induced liver damage in rodent models, underscoring the importance of targeting this pathway [127].

In addition to propagating hepatotoxicity, EVs also modulate immune responses during DILI. Hepatocytes exposed to toxic drugs release EVs carrying DAMPs, such as high-mobility group box 1 and heat shock proteins, which activate innate immune cells and perpetuate inflammatory cascades [128]. This immune dysregulation contributes to systemic inflammation and multi-organ dysfunction syndrome in severe cases of DILI. A study by Lee et al. revealed that DAMPs-enriched EVs enhance the recruitment of neutrophils and macrophages to the liver, exacerbating tissue damage [116].

EVs also serve as potential biomarkers for the early detection and monitoring of DILI. Studies have identified specific EV cargoes, such as miR-122, miR-192, and cytosolic enzymes [e.g., alanine aminotransferase (ALT) or aspartate aminotransferase (AST)], which reflect the extent of hepatocyte injury and predict treatment response [129]. The unique protein signatures and RNA profiles associated with EVs provide critical insights into the underlying disease mechanisms and offer opportunities for personalized medicine approaches in managing DILI [127].

Recent advances in proteomic profiling and single-vesicle tracking have deepened our understanding of EV-mediated processes in DILI. For example, investigations by Royo et al. revealed that acetaminophen exposure alters the cargo of hepatocyte-derived EVs, leading to changes in the expression of genes involved in lipid handling and energy metabolism [130]. These EVs may transfer toxic metabolites, such as N-acetyl-p-benzoquinone imine, to neighboring cells, propagating oxidative stress and mitochondrial dysfunction. Additionally, the interaction between EVs and systemic inflammatory networks shapes the severity of DILI caused by drugs like acetaminophen or statins, emphasizing the need for comprehensive investigations into their functional consequences [127].

### 5.4. EVs in HCC

HCC, which is a leading cause of cancer-related mortality worldwide, often arises in the context of chronic liver diseases such as MASLD, viral hepatitis, and cirrhosis. EVs play a multifaceted role in HCC pathogenesis by modulating the tumor microenvironment, promoting angiogenesis, facilitating immune evasion, and driving metastasis. Their ability to transport oncogenic cargoes enables them to influence both local and systemic effects during HCC progression. Among the various cargoes, the most important include caspase-3, miR-122, miR-21, miR-221, heat shock protein 70, TGF-β, ceramides, long non-coding RNAs, and PD-L1. These cargoes play critical roles in driving tumor growth, immune evasion, angiogenesis, and metastasis, underscoring the pivotal role of EVs in HCC pathogenesis [131].

Tumor-derived EVs significantly contribute to tumor microenvironment modulation by delivering pro-angiogenic factors, such as vascular endothelial growth factor (VEGF) and fibroblast growth factor, which stimulate neoangiogenesis and enhance nutrient supply to the tumor [132]. For instance, Cheng et al. demonstrated that HCC-derived EVs enriched with VEGF increased capillary formation in rodent models, underscoring their role in tumor vascularization [133]. Additionally, EVs derived from cancer-associated fibroblasts deliver MMPs and other proteases that degrade the ECM, facilitating tumor cell migration and invasion [134].

Immune evasion is another critical mechanism mediated by EVs in HCC progression. Tumor-derived EVs carry immunosuppressive molecules, such as PD-L1 and TGF-β, which dampen antitumor immunity and promote tumor growth. These EVs suppress cytotoxic T-cell activity and polarize macrophages toward an M2 phenotype, creating an immunosuppressive microenvironment conducive to tumor survival [135]. Emerging research by Liu et al. suggests that neutralizing PD-L1-enriched EVs restores antitumor immunity and enhances the efficacy of immune checkpoint inhibitors in preclinical models [136].

**Figure 6:**
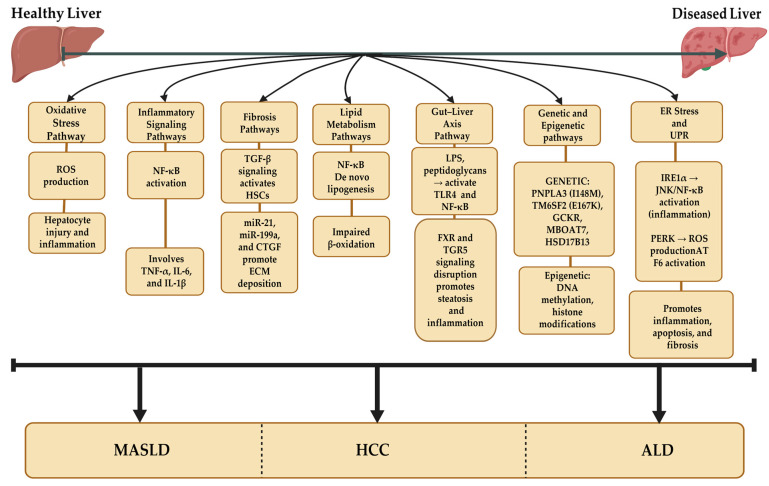
Pathological pathways involved in liver diseases. FXR: farnesoid X receptor; GCKR: glucokinase regulatory protein; HSD17B13: hydroxysteroid (17-beta) dehydrogenase 1; IRE1α: inositol-requiring enzyme 1 alpha; JNK: c-Jun N-terminal kinase; LPS: lipopolysaccharide; MBOAT: membrane-bound O-acyltransferase; NF-κB: nuclear factor kappa-light-chain-enhancer of activated B cells; PERK: protein kinase R (PKR)-like endoplasmic reticulum kinase; PNPLA3: patatin-like phospholipase domain-containing protein 3; ROS: reactive oxygen species; TGR5: Takeda G-protein-coupled receptor 5; TLR4: Toll-like receptor 4; TM6F2: Transmembrane 6 superfamily member 2.

Metastasis represents a major challenge in HCC management, and EVs play a pivotal role in preparing pre-metastatic niches in distant organs. Tumor-derived EVs transfer oncogenic cargoes, such as miR-21 and Twist, which reprogram recipient cells to support tumor colonization [137]. Zhou et al. demonstrated that HCC-derived EVs carrying miR-21 enhance epithelial-to-mesenchymal transition in recipient cells, promoting metastasis to the lungs and lymph nodes [138]. This process is closely linked to HSCs activation and ECM remodeling, further amplifying tumor progression. EV-based liquid biopsies represent a promising tool for early detection and prognosis prediction in HCC. Specific EV cargoes, such as glypican-3, alpha-fetoprotein, and miR-223, reflect the extent of tumor burden and predict treatment response. Recent studies have shown that monitoring changes in EV cargoes over time offers real-time insights into disease progression and treatment efficacy, enabling timely interventions and improving patient outcomes [139]. A schematic summary of EV-mediated pathways across all liver diseases is shown in Figure 6.

## 6. Biomarkers and Therapeutic Potential

### 6.1. EVs as Biomarkers

EVs have emerged as powerful diagnostic and prognostic tools for MASLD and other liver diseases because of their ability to reflect molecular changes occurring within the liver microenvironment. These vesicles carry a diverse array of cargoes, including miRNAs, proteins, lipids, and metabolites, offering tissue-specific insights into disease initiation, progression, and treatment response. Unlike traditional markers, such as ALT and AST, which lack specificity, EV cargoes provide precise information about hepatic alterations at various stages of disease.

In MASLD, specific EV cargoes, such as miR-122 and glypican-3, serve as valuable biomarkers for early detection and disease staging. MiR-122, a liver-specific microRNA involved in regulating lipid metabolism, has been consistently linked to increased hepatic fat accumulation, inflammation, and fibrosis progression. Wang et al. demonstrated that plasma-derived EVs carrying miR-122 strongly correlate with liver histology scores, highlighting its potential as an indicator of disease severity [128]. Similarly, glypican-3-enriched EVs reflect hepatocyte injury levels and predict fibrosis stages, making them critical for monitoring disease progression [140]. EVs derived from lipotoxic hepatocytes play a pivotal role in driving fibrogenesis in MASLD. These EVs induce a phenotypic switch in HSCs, transitioning them from a quiescent state to an activated, profibrotic state [141]. For instance, Povero et al. identified miR-128-3p as a key cargo in these EVs, which targets PPARγ and promotes fibrogenesis [91]. Additionally, Lee et al. demonstrated, through microarray analysis, that lipotoxic hepatocytes release EVs enriched with significantly elevated levels of miR-122 and miR-192, both of which are implicated in MASLD development [142]. Notably, miR-192 upregulates fibrosis markers and enhances the profibrogenic activity of HSCs. Elevated levels of miR-122 and miR-192 are also observed in patients with advanced-stage MASLD compared to those with early-stage disease [141]. More recently, Luo et al. identified miR-1297 in lipotoxic hepatocyte-derived EVs, showing its ability to activate HSCs via the phosphatase and tensin homolog pathway [143].

Beyond MASLD, EV-based biomarkers hold promise for diagnosing and managing other liver diseases. In viral hepatitis, EVs carrying viral components, such as HBV DNA or HCV RNA, indicate viral load and treatment efficacy. For example, HBV-DNA-containing EVs reflect antiviral therapy responses, enabling personalized adjustments [144]. In ALD, EVs enriched with oxidative stress markers, such as malondialdehyde and 4-hydroxynonenal, provide insights into ethanol-induced liver damage and fibrosis development [145]. Longitudinal profiling of EV cargoes allows for real-time monitoring of disease activity and treatment response, facilitating timely interventions. Specific miRNAs, such as miR-192, correlate with fibrosis stage in MASLD patients, while alpha-fetoprotein and des-gamma-carboxy prothrombin enable the early detection of HCC in high-risk populations. Advances in single-vesicle analysis and multi-omics integration enhance the sensitivity and specificity of EV-based diagnostics, paving the way for precision medicine approaches [146].

Despite these advancements, challenges remain in standardizing isolation methods and analytical platforms for reliable clinical applications. Variability in sample processing, storage conditions, and detection techniques necessitates robust protocols and large-scale validation studies [147]. Overcoming these hurdles will further solidify the translational relevance of EV-based biomarkers in hepatology.

The key EV-associated biomarkers relevant to MASLD and other liver diseases are detailed in Table 3.

### 6.2. Therapeutic Strategies Targeting EV-Mediated Pathways

EVs represent promising therapeutic targets in MASLD and related liver diseases because of their central role in mediating intercellular communication, inflammation, fibrosis, and tumor progression. Targeting EV-mediated pathways offers diverse strategies for mitigating disease progression, restoring hepatic homeostasis, and enhancing treatment efficacy. These approaches include modulating EV cargo composition, inhibiting EV biogenesis or uptake, engineering EVs for drug delivery, and neutralizing specific EV cargoes involved in pathogenic processes.

One of the primary therapeutic strategies involves modulating EV cargo composition to deliver therapeutic molecules directly to target cells. For instance, EVs can be engineered to carry small interfering RNAs (siRNAs), miRNAs, or proteins that suppress pro-inflammatory, profibrotic, or pro-tumorigenic signaling. Li et al. demonstrated that administering EVs enriched with miR-29 significantly reduces collagen deposition and improves liver function in rodent models of MASLD-related fibrosis [90]. Similarly, siRNA-loaded EVs targeting β-catenin or TGF-β signaling have shown promise in suppressing oncogenic pathways in HCC [148]. Pharmacological inhibition of EV biogenesis or uptake represents another viable strategy for managing liver diseases.

Compounds such as GW4869, which blocks neutral sphingomyelinase activity, and dynasore, which disrupts endocytosis, reduce the propagation of pro-inflammatory and profibrotic signals mediated by EVs. Sun et al. demonstrated that inhibiting EVs release using GW4869 alleviates hepatic inflammation and fibrosis in patients with NASH. Furthermore, blocking EVs uptake by recipient cells using competitive inhibitors or receptor antagonists prevents the transfer of toxic cargoes, such as ROS or lipotoxic intermediates, thereby reducing cellular damage and improving outcomes [149].

Engineering EVs for targeted drug delivery addresses limitations of conventional therapies by leveraging their natural ability to cross biological barriers and deliver cargo selectively to recipient cells. For example, mesenchymal stem cell-derived EVs loaded with superoxide dismutase effectively mitigate oxidative stress and improve liver histology in rodent models of ALD [150,151]. Similarly, EVs carrying interferon-alpha have been explored as antiviral agents in chronic HBV infection, offering a nontoxic alternative to nucleoside analogues [152]. Beyond traditional therapeutic cargoes, emerging research highlights the potential of engineered EVs delivering CRISPR/Cas9 systems for antiviral therapies. These EVs can be designed to target viral genomes directly, such as disrupting HBV covalently closed circular DNA (cccDNA) or silencing HCV RNA replication, thereby suppressing viral persistence and restoring host immune responses [153]. Neutralizing specific EV cargoes disrupts key mediators of disease progression, holding significant therapeutic potential. Antibodies targeting pro-inflammatory cytokines, such as TNF-α or IL-1β, or immunomodulatory molecules, such as PD-L1, reduce inflammation and restore antitumor immunity in HCC and viral hepatitis [154]. Additionally, inhibiting the transfer of oncogenic cargoes, such as miR-21 or Twist, via EVs suppresses metastasis and enhances treatment response in HCC [155].

Emerging research explores innovative applications of EVs, such as EV-based vaccines for chronic viral hepatitis. These vaccines, carrying viral antigens like HBV surface antigen or HCV core protein, induce robust immune responses without the risks associated with live attenuated viruses [156]. Reynolds et al. demonstrated that EV-based vaccines protect against HBV reactivation in chronically infected individuals, highlighting their potential for long-term disease management [157]. Despite these advancements, challenges persist in translating EV-based therapies into clinical practice. Variability in isolation methods, cargo loading efficiency, and biodistribution affects therapeutic efficacy and safety. Standardization of protocols and validation across large cohorts are essential for ensuring reliable outcomes. Moreover, ethical considerations surrounding engineered EVs require careful evaluation, particularly regarding off-target effects and regulatory compliance [23]. Efforts to optimize delivery systems, enhance specificity, and reduce costs will further accelerate the adoption of EV-based therapies in hepatology.

### 6.3. Challenges and Future Directions

While EVs hold immense promise as biomarkers and therapeutic targets in MASLD and other liver diseases, several challenges must be addressed to fully realize their potential. Indeed, the diagnostic use of EVs for the management of liver diseases is still at the beginning. These challenges span technical, clinical, and regulatory domains, requiring interdisciplinary efforts and innovative solutions.

One of the primary technical challenges emerging from the studies conducted so far involves standardizing methods for isolating and characterizing EVs. Variability in sample processing, storage conditions, and detection techniques significantly affects the reliability and reproducibility of EV-based diagnostics and therapeutics. For instance, differences in centrifugation speeds, ultracentrifugation protocols, or commercial kits used for EVs isolation often lead to inconsistent results across studies [158]. Hartjes et al. emphasized the need for establishing robust and universally accepted protocols to ensure consistency in EVs research. Furthermore, advancements in analytical platforms, such as NTA, tunable resistive pulse sensing, and mass spectrometry, are essential for accurately quantifying and profiling EV cargoes [24]. In addition, the use of techniques to obtain engineered EVs could be helpful in overcoming this bias since those EVs have been reported to be very effective in delivering therapeutic agents with improved targeting and reduced side effects [159].

Another challenge lies in understanding the heterogeneity in EVs populations and their functional diversity. EVs originate from various cell types within the liver microenvironment, including hepatocytes, KCs, HSCs, and immune cells, each carrying distinct cargoes with unique biological effects [160]. Deciphering the specific roles of different EVs subpopulations in disease pathogenesis requires advanced tools and multi-omics approaches. For example, integrating transcriptomic, proteomic, and lipidomic analyses could provide comprehensive insights into the molecular signatures of EVs associated with specific stages of MASLD or other liver diseases [161]. Such integrative strategies will enhance our ability to identify disease-specific EVs biomarkers and develop targeted therapies. Also, there is a great heterogeneity in EVs among various subjects based on age, gender, and physio/pathological conditions. For this reason, establishing standardized parameters to differentiate healthy controls from patients can be a great challenge [162]. Finally, the sensitivity and specificity of diagnostic EVs for liver disease detection would require large cohorts of patients in clinical trials, which is mandatory for precise diagnosis.

The clinical translation of EV-based diagnostics and therapeutics also faces significant hurdles. Firstly, the use of methods like Principal Component Analysis, t-Stochastic Neighbor Embedding, Uniform Manifold Approximation and Projection, Support Vector Machine, deep learning, nanoplasmonics, electrical interfaces, signal analysis, cryo-transmission electron microscopy, and atomic force microscopy, which can be applied to integrate different types of omics data, such as genomics, transcriptomics, proteomics, and metabolomics, and add details about the physical characteristics and biomechanical properties of EVs, could be useful to provide a more comprehensive understanding of the complexity of EVs. In this way, it could be more feasible to translate the use of EVs into an advanced precision drug delivery [163,164,165].

Despite promising preclinical findings, large-scale validation studies are needed to confirm the efficacy and safety of EV-based interventions in diverse patient populations. For instance, the administration route, dosage, and adverse effects of EVs should be properly evaluated in clinical trials to broaden their clinical utilization. Moreover, an improvement in the knowledge about several aspects related to the therapeutic use of EVs is needed. Among various aspects, issues related to stable storage and transportation of EVs, EV production quality and control, more precise and effective methods for large-scale production and purification of EVs, and the criterion for choosing the proper administration route and dosage, should be addressed [90]. For those reasons, and in order to translate the use of EVs into a clinical scenario, worldwide conferences should be organized with the purpose of conveying technical issues into practice. Ethical considerations surrounding the use of engineered EVs, particularly regarding off-target effects and long-term consequences, require careful evaluation [166]. Regulatory frameworks governing the development and approval of EV-based products must evolve to accommodate the unique properties of these nanoscale mediators. Efforts to establish guidelines for quality control, biodistribution, and immunogenicity will facilitate the transition of EV-based technologies from bench to bedside. Emerging technologies offer exciting opportunities for overcoming these challenges and advancing the field of EVs research. Advances in single-vesicle analysis allow for the characterization of individual EVs, providing unprecedented resolution into their cargo composition and functional properties [23]. Similarly, CRISPR/Cas9-based gene editing enables precise manipulation of EV cargo, opening new avenues for therapeutic innovation. For instance, Berggreen et al. demonstrated the feasibility of using CRISPR-edited EVs to deliver antifibrotic miRNAs specifically to HSCs, highlighting the potential of this approach in treating fibrosis-related liver diseases [25].

Future research should focus on elucidating the dynamic changes in EV cargo during disease progression and treatment response. Longitudinal studies tracking EV profiles over time could reveal novel biomarkers indicative of disease activity or therapeutic efficacy [83]. Moreover, exploring the interplay between EVs and other cellular mediators, such as cytokines, exosomes, and MVs, will deepen our understanding of their role in liver pathophysiology. Machine learning algorithms and artificial intelligence-driven analytics may further accelerate the identification of disease-specific EVs signatures and optimize therapeutic strategies [167].

Additionally, the interaction between EVs and systemic inflammatory networks shapes the magnitude of liver injury, underscoring the need for comprehensive investigations into their functional consequences. Recent evidence suggests that specific EV cargoes, such as integrin and adhesion molecules, facilitate the attachment of HSCs to ECM scaffolds, enhancing their activation and proliferation [168]. Similarly, the transfer of specific lipids via EVs alters membrane fluidity in recipient cells, promoting fibrotic signaling pathways. These findings highlight the complexity of EV-mediated mechanisms and emphasize the need for detailed mechanistic studies.

To fully realize the potential of EVs in diagnosing and treating liver diseases, interdisciplinary approaches that integrate advanced technologies with traditional methodologies are essential. One promising avenue is the application of artificial intelligence (AI) to analyze EV cargoes and optimize their use in precision medicine. AI-driven tools can process vast datasets generated by multi-omics analyses of EVs, enabling the identification of disease-specific biomarkers and the prediction of treatment responses with unprecedented accuracy. For instance, Greenberg et al. proposed an innovative framework for integrating AI into the design and optimization of EV-based drug carriers, emphasizing the ability of AI to predict optimal cargo loading, biodistribution, and therapeutic efficacy. By leveraging AI models, researchers can simulate how specific modifications to EV cargoes—such as CRISPR/Cas9 components or anti-inflammatory miRNAs—impact their interactions with target cells and tissues [163].

Addressing the challenges in standardizing EV isolation methods and resolving heterogeneity issues will enhance their translational relevance. Efforts to develop scalable, cost-effective isolation techniques and validate findings across large cohorts are essential for ensuring reliable outcomes. Moreover, ethical considerations surrounding the use of engineered EVs must be carefully evaluated, particularly in terms of off-target effects and regulatory compliance [158]. Establishing standardized protocols and guidelines will further solidify the clinical utility of EV-based technologies.

## 7. Conclusions

In conclusion, EVs have become a cornerstone in hepatological research, offering profound insights into the pathogenesis of MASLD and other liver diseases. By transporting a wide array of cargoes—including miRNAs, proteins, lipids, and metabolites—EVs enable precise monitoring of molecular changes within the liver microenvironment, making them invaluable for non-invasive diagnostics and innovative therapeutic strategies. Overcoming current technical challenges, such as standardizing isolation methods and resolving heterogeneity issues, as well as addressing ethical concerns related to engineered EVs, will be critical in enhancing their reliability and safety for clinical applications. This, in turn, will improve patient outcomes and deepen our understanding of liver biology. Moving forward, research efforts should focus on elucidating the intricate and dynamic interactions between EVs and cellular mediators, ensuring the complete realization of their potential in both diagnosis and treatment. Such advancements hold promise for personalized medicine approaches and could revolutionize the management of MASLD and associated conditions.

In order to achieve these goals, large-scale clinical trials specifically investigating the use of EVs to interrupt the occurrence or progression of MASLD should be organized. While preclinical studies have demonstrated the potential of EVs as diagnostic biomarkers and therapeutic tools in MASLD, the translation of these findings into human trials remains limited. This gap highlights a significant unmet need in the field and underscores the challenges associated with developing EV-based interventions for MASLD. Addressing these challenges will require interdisciplinary collaboration, robust funding, and the establishment of standardized protocols for EV isolation, characterization, and application. Additionally, long-term safety evaluations and regulatory frameworks must be developed to ensure the safe and effective implementation of EV-based therapies. By bridging the gap between preclinical discoveries and clinical applications, we can unlock the full potential of EVs as transformative tools in the fight against MASLD and other liver diseases.

## Figures and Tables

**Table 1:** EV biogenesis and cargoes.

EVs Subtype	Size (Diameter)	Key Markers	Biogenesis Pathways	Cargo Types	References
**Exosomes**	30–150 nanometers	CD63, CD81, CD9; nucleic acids (e.g., miRNAs, mRNAs)	Inward budding of endosomal membranes forming intraluminal vesicles in multivesicular bodies; regulated by ESCRT complex, Alix, and Tsg101	Proteins (e.g., tetraspanins), lipids, nucleic acids, metabolites.	[26,27,28,29]
**MVs**	100–1000 nanometers	Influenced by ADAM10 and ADAM17 enzymes	Direct outward budding from plasma membrane; driven by cytoskeletal dynamics, calcium levels, and enzymatic activities	Larger cytoplasmic fragments, organelles, lipids (e.g., ceramides), pro-inflammatory cytokines (e.g., TNF-α, IL-1β)	[30,31,32,33]
**Apoptotic Bodies**	500–4000 nanometers	Phosphatidylserine, annexin V, thrombospondin, complement protein C3b	Formed during apoptosis; outward blebbing of cell membrane driven by actin-myosin interactions and cellular fragmentation	Cellular debris, nuclear chromatin, proteins; associated with cell death and inflammation	[34,35,36]
**Ectosomes**	100–1000 nanometers	Not specified	Outward budding of plasma membrane; involves reorganization of membrane and cytoskeletal components	Implied to be distinct from exosomes or microvesicles, potentially including unique proteins and lipids	[37]
**Exomeres**	<50 nanometers	Unique proteomic profiles	Nonvesicular nanoparticles; biogenesis not fully described, but distinct from vesicular EVs	Proteins with specialized profiles; involved in intercellular communication and disease progression	[38]
**Supermeres**	<50 nanometers	Enriched with RNAs	Nonvesicular nanoparticles; biogenesis mechanisms unclear, but associated with enhanced tissue accumulation	RNAs (highly enriched), disease biomarkers, therapeutic targets; exhibits biodistribution patterns for intercellular signalling	[38]

ADAM10: A disintegrin and metalloproteinase domain-containing protein 10; CD: cluster of differentiation; ESCRT: endosomal-sorting complex required for transport; EVs: extracellular vesicles; IL: interleukin; MVs: macrovesicles; RNA: ribonucleic acid; TNF-α: tumor necrosis factor alpha.

**Table 2:** EV genetic cargoes in MASLD.

Gene	Variant	Prevalence Rate	Functional Consequences	Clinical Implications for MASLD Prevention/Control	Reference
***PNPLA3***	I148M (rs738409)	~20–40% globally	Reduces triacylglycerol hydrolysis, leading to lipid accumulation, inflammation, and fibrosis progression.	Carriers are at higher risk for severe liver disease; lifestyle modifications (e.g., calorie restriction, exercise) may mitigate risk.	[39]
***TM6SF2***	E167K (rs58542926)	~5–10% globally	Impairs very-low-density lipoprotein secretion, increasing hepatic fat content but reducing circulating lipids and cardiovascular risk.	Personalized dietary and pharmacological interventions targeting lipid export pathways could reduce hepatic steatosis.	[42]
***GCKR***	P446L (rs1260326)	~20–30% globally	Enhances glucokinase activity, promoting insulin sensitivity but upregulating de novo lipogenesis.	Genotype-guided dietary strategies (e.g., low-carbohydrate diets) may help manage lipid synthesis and reduce hepatic fat accumulation.	[43]
***MBOAT7***	rs641738	~10–20% globally	Alters phospholipid remodelling, leading to altered lipid composition and increased inflammation.	Targeting phospholipid metabolism pathways may offer therapeutic potential to reduce inflammation and fibrosis risk.	[45]
***HSD17B13***	rs72613567	~5–15% globally	Protective effects against MASLD progression by reducing inflammation and fibrosis risk.	Understanding protective mechanisms could guide the development of anti-inflammatory therapies for MASLD.	[45]

GCKR: glucokinase regulatory protein; MASLD: metabolic dysfunction-associated steatotic liver disease; *PNPLA3:* patatin-like phospholipase domain-containing protein 3; *TM6SF2:* transmembrane 6 superfamily member 2.

**Table 3:** EV-associated biomarkers in MASLD and other liver diseases.

Liver Disease	EV-Associated Biomarker	Significance	Reference
**MASLD**	miR-128-3p	Promotes fibrogenesis by targeting PPARγ in HSCs	[91]
**MASLD**	miR-122	Early detection, disease staging, correlates with liver histology scores	[128]
**MASLD**	Glypican-3	Reflects hepatocyte injury levels, predicts fibrosis stages	[140]
**MASLD**	miR-192	Upregulates fibrosis markers, enhances profibrogenic activity of HSCs	[141]
**MASLD**	miRNA-1297	Activates HSCs via the PTEN pathway	[143]
**Viral Hepatitis**	HBV DNA/HCV RNA	Indicates viral load, reflects antiviral therapy response, aids in personalized treatment adjustments	[144]
**ALD**	Oxidative Stress Markers (e.g., Malondialdehyde, 4-Hydroxynonenal)	Provides insights into ethanol-induced liver damage and fibrosis development	[145]
**HCC**	Alpha-fetoprotein, Des-gamma-carboxy prothrombin	Enables early detection of HCC in high-risk populations	[146]

ALD: alcoholic liver disease; DNA: deoxyribonucleic acid; EVs: extracellular vesicles; HBV: hepatitis B virus; HCC: hepatocellular carcinoma; HSC: hepatic stellate cell; MASLD: metabolic dysfunction-associated steatotic liver disease; miR: microRNA; PPAR-γ: peroxisome proliferator-activated receptor gamma; PTEN: phosphatase and tensin homolog; RNA: ribonucleic acid.
